# The Impact of a City-Level Minimum Wage Policy on Supermarket Food Prices by Food Quality Metrics: A Two-Year Follow Up Study

**DOI:** 10.3390/ijerph16010102

**Published:** 2019-01-01

**Authors:** James Buszkiewicz, Cathy House, Anju Aggarwal, Mark Long, Adam Drewnowski, Jennifer J. Otten

**Affiliations:** 1 Epidemiology, Center for Public Health Nutrition, University of Washington, Seattle, WA 98195, USA; buszkiew@uw.edu (J.B.); anjuagg@uw.edu (A.A.); adamdrew@uw.edu (A.D.); 2 Nutritional Sciences Program, University of Washington, Seattle, WA 98195, USA; catherineehouse@gmail.com; 3 Daniel J. Evans School of Public Policy and Governance, University of Washington, Seattle, WA 98195, USA; marklong@uw.edu; 4 Environmental and Occupational Health Sciences, Center for Public Health Nutrition, University of Washington, Seattle, WA 98195, USA

**Keywords:** minimum wage, market basket, food cost, supermarkets, food price

## Abstract

*Objective*: To examine the effects of increasing minimum wage on supermarket food prices in Seattle over 2 years of policy implementation, overall and differentially across food quality metrics. *Methods:* Prices for the UW Center for Public Health Nutrition (CPHN) market basket of 106 foods were obtained for 6 large supermarket chain stores in Seattle (“intervention”) and for the same chain stores in King County (“control”) at four time points: 1-month pre- (March 2015), 1-month post- (May 2015), 1-year post- (May 2016), and 2-years post-policy implementation (May 2017). Prices for all food items were standardized and converted to price per 100 kcal. Food quality metrics were used to explore potential differential price increases by (a) food groups, as defined by US Department of Agriculture; (b) NOVA food processing categories, and (c) nutrient density quartiles, based on the Nutrient Rich Foods Index 9.3. Separate difference-in-differences linear regression models with robust standard errors, examined price differences per 100 kcal overall, clustered by store chain, and stratified by each food quality metric. *Results:* There were no overall market basket price changes attributable to Seattle’s minimum wage policy. Moreover, no minimum wage effect was detected by USDA food group, food processing, or nutrient density categories. *Conclusions:* Local area supermarket food prices were not impacted by Seattle’s minimum wage policy 2 years into policy implementation and after the first increase to $15/h overall or by sub-classification. Low-income workers may be able to afford higher quality diets if wages increase yet supermarket prices stay the same.

## 1. Introduction

The federal minimum wage rate in the United States has not kept pace with inflation since 1968 contributing to growing wage inequities and a decline in purchasing power among low- and minimum-wage earning workers and their families [1,2]. Recently, in an effort to improve the economic environment of workers and increase the economic security and well-being of workers and their families, an increasing number of municipalities and counties have adopted wage rates above that of their state [3]. In 2003, Santa Fe, New Mexico and San Francisco, California became the first cities to adopt such local minimum wage ordinances [4] and, in 2014, Seattle became the first city to adopt a $15/h minimum wage [5]. On January 1st, 2017, many low wage workers in the City of Seattle saw their first minimum wage increase to $15/h [5].

While proponents of higher minimum wages argue that such increases are necessary to improve purchasing power and combat stagnating wages, there is concern that increased labor costs, which comprise, on average, one-third of total costs, will lead to a rise in the cost of goods, potentially offsetting these benefits for low-wage workers [6,7,8]. Increases in the cost of food are of particular concern given that one-third of all low-wage workers are employed in the food system and because low- and minimum wage workers spend a larger share of their income on food [9].

Limited evidence is available on the effect of minimum wage policies on food prices, particularly grocery store prices, and even fewer studies perform analyses to assess if there might be differential price increases on more nutritious food items [7,10,11,12,13,14]. Evaluations of minimum wage effects on food prices in the restaurant and fast food industries demonstrate pass-through effects ranging from 0.56% to 4% based on the timing and magnitude of the minimum wage increase (10% to 33%) [7,10,11,12]. However, our prior research on the early effect of a local minimum wage ordinance has found little effect on grocery store food prices at 1-month (16% increase, $9.47 to $11) or 1-year (37% increase, $11 to $13) post-policy implementation either overall, by food group, or by level of food processing [13,14].

Understanding the potentially differential price effects of minimum wage increases is important given that higher cost of healthy foods has been proposed as one of the underlying barriers to healthy eating, particularly among lower socioeconomic groups [15,16,17,18,19]. If increased labor costs due to minimum wage do not lead to an increase in food prices, then minimum wage policies may help low-wage workers achieve higher quality diets through greater food purchasing power. By contrast, if higher labor costs due to minimum wage increases cause a rise in food prices overall or differentially by nutritional content (e.g., more nutritious or nutrient-dense foods rise in cost faster than less nutritious foods), then low-wage workers may experience a decline in purchasing power for healthy foods, ultimately leading to poorer health. Low- or minimum-wage earning shoppers are significantly more price sensitive than their higher income counterparts [14,19,20]. Extant research has shown that policies or interventions that tax, such as recent city-level soda taxes [21], or target discounts on certain healthy foods, such as the United States Department of Agriculture’s Healthy Incentives Pilot [22], can influence shopping behavior and consumption.

In this analysis, we examine differential price effects across three food quality metrics: food group, level of food processing, and nutrient density. These metrics were chosen because they have been associated with health outcomes and because they exhibit heterogeneity by food price when comparing across metrics [17,19,21,23,24,25,26,27,28,29,30,31]. The Centers for Disease Control and Prevention efforts to improve diet have focused on increasing the consumption of fruits and vegetables [25]. However, fruits and vegetables have been shown to be more expensive than other food groups. Thus, researchers and policymakers have explored pricing strategies, either through discounts or taxes, to encourage (e.g., fruit) or discourage (e.g. sugar-sweetened beverages) the consumption of certain food groups [21,26]. Consumption of ultra-processed food items have been linked to increased risk of certain chronic diseases, such as obesity, as well as increased consumption of added sugars [27,28,29,30]. Moreover, the convenience and pricing of processed and ultra-processed foods at supermarkets has been shown to influence their consumption [31]. Several studies have provided evidence that prices vary across the nutrient density profiles of food items with foods low in key micronutrients and high in energy (e.g., sugar sweetened beverages) being among the least expensive choices available to consumers [19]. Consumption of these low-nutrient foods has been linked to higher risk of obesity and there is concern that more price-sensitive populations, such as low-wage earners and their families, may consume such food items more frequently and thus may be at greater risk of developing diet-related chronic disease [17,23,24].

The present study has several strengths and builds on our prior work in several key ways [13,14]. Previously, we provided an illustration of methods for the application of a market basket data collection tool to examine changes in food prices for localities interested in tracking and comparing the price effects of similar policy changes. As with this paper, our prior work evaluated potential price effects overall as well as by food group and level of food processing, however, our prior work evaluated impacts at fewer time points and prior to fuller implementation of the Seattle Minimum Wage Ordinance. In the current analysis, we build upon these insights by adding a new food quality metric to access the potential for differential food price increases by nutrient density using the Nutrient Rich Foods Index 9.3 [32]. In addition, rather than standardizing food prices by weight or volume, as we have in our previous publication, we evaluate food price per 100 kcal. This standardized price measure allows us to evaluate changes across food group, food processing, and nutrient density strata in a way that is more reflective of human behavior and choices at the point of purchase. That is, when low-wage-earning shoppers make food purchasing decisions they are more likely to have incentive to obtain the greatest number of calories for the lowest price rather than the greatest weight of food. Finally, this analysis evaluates the effect of both an additional year of exposure to the Seattle Minimum Wage Ordinance as well as an additional increase in the policy phase-in wage rate, from $12.50–$13 per hour to $13.50–$15 per hour. This is of key importance as this paper is the first evaluation of the effect of a minimum wage increase to $15 per hour on local area supermarket food prices.

## 2. Methods

### 2.1. Study Design

In June 2014, the Seattle City Council adopted legislation which phased the City of Seattle’s minimum wage rate to $15/h variably between 2015 and 2019–2021 [5,33]. The phase-in schedule is driven by both the size of the business and whether the business contributes to health insurance benefits for its workers [5,34]. As of its initial phase-in on 1 April 2015, the city’s minimum wage increased from $9.47/h to $11/h for most large (≥ 500 employees nationally) and some small businesses (<500 employees) and $10/h for other small businesses. On 1 January 2016, the city’s minimum wage increased from $10–$11/h to $12.50–$13/h for large businesses and $10.50–$12/h for small businesses (<500 employees). On 1 January 2017, the city’s minimum wage increased again to $13.50–$15/h for large businesses or $11–$13/h for small businesses. For the purposes of this analysis, all supermarket store chains sampled follow the large employer schedule (see Appendix Table A1).

The specifics of store selection, data collection, and the data collection instruments for this project have been described elsewhere [13,14,33,35]. In brief, data was collected from six supermarkets in Seattle and affected by the ordinance (“intervention”), and six same-chain supermarkets located in King County, but outside of Seattle and unaffected by the ordinance (“control”). We selected store chains that were the most frequently patronized by a representative sample of Seattle-King County residents, had locations in both Seattle and King County, and represented a range of prices from low to high. The six chains included in the sample were representative of 50 out of the 78 individual Seattle stores impacted by the ordinance at the time of its initial implementation in April 2015. Specific store locations were selected based on their proximity to low-income neighborhoods, based on American Community Survey data [36].

Data on 106 food and beverage items were collected using the University of Washington’s (UW) Center for Public Health Nutrition (CPHN) market basket. The UW CPHN market basket, developed in 2009, is a combined and condensed version of the Consumer Price Index and United States Department of Agriculture’s Thrifty Food Plan market baskets and has been enhanced to contain commonly consumed items and nutrient-rich foods and beverages [13,14,33,35,36]. This market basket tool has been used and validated in Seattle stores in a number of prior studies and is described in detail in Otten et. al (2017); it has the advantage of containing more healthy food items needed for a high quality diet [13,14,33,35,36]. Stores were visited at four times points: phase 1 (prior to policy implementation, March 2015), phase 2 (1-month post policy implementation, May 2015), phase 3 (after the phase-in to $13/h and seasonally matched to phase 2, May 2016) and phase 4 (after the phase-in to $15/h and seasonally matched to phase 2 and 3, May 2017) (see Appendix Table A1). At each visit, a trained researcher recorded the lowest non-sale price, in United States dollars (USD), for the identical or comparable item from prior visits. When multiple sizes were available for the same food item, the medium-sized item was selected. Following data collection, prices were rechecked for any anomalies, and any missing or anomalous items were rechecked at the store. 

All prices were standardized to the price per 100 grams and then price per 100 kilocalories (kcal) using the United States Department of Agriculture (USDA) food database [37]. This standardization was used as it was thought to better reflect the purchasing logic of lower income shoppers, obtaining the adequate calories for an affordable price, rather than a focus on the volume of a product. This is particularly relevant when thinking about the quantity of certain low kcal, high cost items such as fresh produce. For example, 100 g of fresh cantaloupe is a very different quantity than 100 kcal. Other methods for standardizing prices include price per 100 grams and price per serving [38], among others; however, these other standardization methods are beyond the scope of this analysis.

### 2.2. Food Categorizations

Once food price data were collected and processed, each food and beverage item was assigned to a food group, a food processing category, and a Nutrient Rich Food Index 9.3 (NRF_9.3_) quartile. Food group categorizations were based on seven food groups defined by the USDA and included “cereals and grains”, “fruits”, “vegetables”, “dairy”, “meats, beans, and proteins”, “fats and oils”, “sugars” and “other beverages” [39]. However, the category “other beverages” was excluded from this analysis as 1) this category contained few items, 2) since each item (coffee, malt beverages, wine) in this category had a high price to kcal ratio, these items represented outliers, and 3) these items are not covered by government programs for which many low-income families would be eligible, such as the Supplemental Nutrition Assistance Program and Women, Infants, and Children.

Food processing level categories were based on the degree to which foods remained in their natural state or underwent alteration to increase shelf life, improve flavor, or make them readily consumable. We used the NOVA food processing classification scheme described in Martínez Steele, et al. (2016), which classifies food items as unprocessed or minimally processed, processed culinary ingredients, processed foods, and ultra-processed food (see Appendix Table A2) [27]. Two researchers independently classified each food item according to this classification scheme and had agreement on ninety-three (90%) of the total 103 food items. A third researcher helped to classify the remaining ten (10%) food items. 

Nutrient quality was assessed using the NRF_9.3_ per 100 kcal, which calculates a nutrient density score for a food item based on the proportion of certain positive and negative macro- and micronutrients contained within [32,40,41]. To calculate the NRF_9.3_, we first obtained the nutrient composition per 100 grams for each food item by linking the CPHN market basket to the Minnesota Nutrition Composition and the USDA SR 28 databases [42]. This included information on total calories along with information on nine nutrients to encourage (protein, fiber, vitamins A, C, and D, calcium, iron, potassium, and magnesium) and three nutrients to limit (added sugars, saturated fat, sodium) in a healthy diet (see Appendix Table A3) [32,40,41]. The NRF_9.3_ utilizes daily value reference quantities established by the Food and Drug Administration and the USDA’s Healthy Eating Index [32,40,41]. For each item in the market basket, the NRF_9.3_ score was calculated using the formula [32,40,41]:

(1) $$NRF_{9.3}=(\sum_{1-9}\frac{\frac{Nutrient_{i}}{DV_{i}}}{ED}-\;\sum_{1-3}\frac{\frac{Li}{MRV_{i}}}{ED})\;\times 100$$

where each of the nine nutrients to encourage (*Nutrient*) and three nutrients to limit (*Li*), per 100 kcal, is divided by the daily reference value to obtain the percent daily value (*DV*) or maximum recommended values (*MRV*), respectively. Each estimate is then divided by the energy density (*ED*) of the given food item, summed with category (encourage, *Nutrient*, or limit, *Li*), and then estimate for nutrients to limited are subtracted from those to encourage for each food item. This value is then multiplied by 100% to obtain the NRF_9.3_ score. To avoid overly inflated NRF_9.3_ scores for low energy density items with extremely high relative amounts of a single nutrient (e.g. green peppers), individual nutrient %DVs that exceeded 100% were top-coded to 100%. Food items were then assigned to one of four quartiles based on NRF_9.3_ score quartiles with quartile 1 comprising the least nutrient dense food items and quartile 4 comprising highly nutrient dense food items (see Appendix Table A3).

### 2.3. Statistical Analysis

We used a difference-in-differences (DD) linear regression model to estimate the mean difference in price per 100 kcal between Seattle chain supermarkets and King County chain supermarkets over time. This approach provides the average treatment effect on the treated (Seattle) stores and assumes that the trends for food prices overtime in both treated and control stores in the pre-policy period were parallel. The percentage of missing food items over time was low (1.5% or 75 out of 4944) and therefore, we chose to conduct a complete case analysis, excluding those items differentially missing across the period of observation. We also exclude three items that had outlier prices per 100 kcal (wine, coffee, and malt beverages), due to the low caloric value relative to price, leaving 103 items for the final analysis. We used the formula:

(2) $$Price_{ijkt}=\beta_{0}+\;\beta_{1}Seattle_{k}+\gamma_{1}Post1_{t}+\gamma_{2}Post2_{t}+\gamma_{3}Post3_{t}+\;\delta_{1}Post1_{t}\times Seattle_{k}\;+\delta_{2}Post2_{t}\times Seattle_{k}+\delta_{3}Post3_{t}\times Seattle_{k}+\varepsilon_{ijkt}$$

where $Price_{ijkt}$ is the estimated mean price for item i at store j in region k (1 = Seattle, “intervention”, and 0 = King County, “control”), at time t.$\;\beta_{0}$ is the intercept and average price for King County stores at baseline, i.e. prior to policy implementation. $Seattle_{k}$ is an indicator variable that equals 1 for Seattle stores and 0 for King County store, and $\beta_{1}$ captures the mean difference in item-level price between Seattle and King County stores at baseline. $Post1_{t}$, $Post2_{t}$, and $Post3_{t}$ are indicator variables which equal 1 for each seasonally-matched data collection period: follow-up 1 (May 2015), follow-up 2 (May 2016), follow-up 3 (May 2017), respectively, and 0 if otherwise, with the reference period being the pre-policy implementation baseline. $\gamma_{1}$, $\gamma_{2}$, $\gamma_{3}$ are the differences in mean item-level price for each follow-up period post-policy implementation relative to the pre-policy baseline period. $Post1_{t}\times Seattle_{k}$, $Post2_{t}\times Seattle_{k}$, and $Post3_{t}\times Seattle_{k}$ equals 1 for Seattle stores in follow-up period 1, 2, and 3, respectively, relative to baseline, and $\delta_{1}$, $\delta_{2}$, $\delta_{3}$ provide the estimated average policy treatment effect on the treated (Seattle) stores are the coefficients of interest. More specifically, $\delta_{1}$, $\delta_{2}$, $\delta_{3}$ provide the mean difference in item-level price attributable to the minimum wage ordinance in each follow-up period, relative to baseline. $\varepsilon_{ijkt}$ is the residual error term. To examine potential modification of the effect of minimum wage on price by food group, food processing, and nutrient density, separate DD linear regression models (Equation (2), above) were run overall and within each food quality strata—food group, food processing category, and nutrient density quartiles. We did not include additional model controls due the small number of sampled stores; however, region and time fixed effects as well as same-chain store matching across regions likely account for some unobserved confounding. Robust standard errors were clustered to account for food price correlation within stores and an α level of 0.05 was used to determine statistical significance. All analyses were conducted using Stata version 14 [43].

## 3. Results

**Figure 1:**
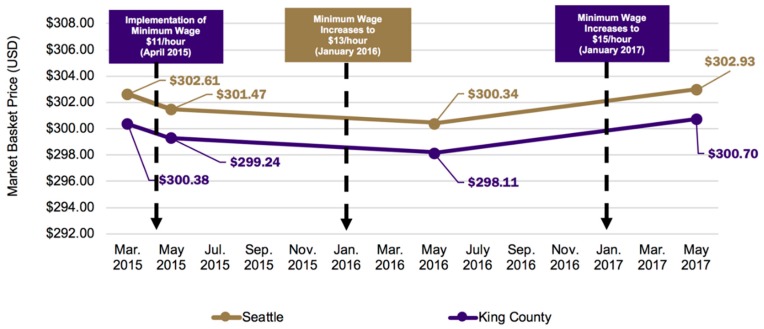
Average market basket price at one-month pre- and at 1-month, 1-year, and 2-years post-implementation of Seattle’s minimum wage in intervention (Seattle) and control (King County) supermarkets. Notes: The minimum wage increase schedule highlighted in the figure follows that for firms with >500 employees worldwide and who do not provide health benefits for their employees.

Figure 1 displays the average total market basket price for Seattle and King County locations, standardized across locations such that each item had the same weight (in grams) or volume (in ounces) across stores, rather than by kcal, at baseline and follow-up periods 1 through 3. While King County stores had a slightly lower average market basket price than Seattle stores, this difference remained relatively constant overtime. This is also reflected in the non-significant, mean difference item-level price per kcal at 1-month (−$0.01 per 100 kcal, SE = 0.026, *P* = 0.670), 1-year ($0.00 per 100 kcal, SE = 0.019, *P* = 0.928), and 2-years ($0.00 per 100 kcal, SE = 0.024, *P* = 0.861) post-policy implementation between Seattle and King County locations (Table 1).

Food group stratified, multi-level DD regression model results are shown in Table 1. While there were significant temporal changes from baseline to follow-up 3 for cereals & grains, and vegetables, as with the overall results, there was no significant effect on food prices per 100 kcal overall or by food group attributable to the Seattle Minimum Wage Ordinance. The largest observed price change by food group was for fruit, which experienced a nonsignificant decline of $0.05 per 100 kcal (SE = 0.09) from baseline to follow-up 3 in Seattle supermarkets relative to King County supermarkets. Fruit, sugar and sweets, and vegetables from baseline to follow-up and sugar and sweets from baseline to follow-up 2 experienced the next largest changes with nonsignificant declines of $0.03 per 100 kcal.

Table 2 displays DD regression results for mean difference in price estimates over time between Seattle and King County supermarkets by level of food processing. There was no significant minimum wage effect on mean difference in price per 100 kcal across any food processing category strata. The largest observed price change by level of food processing in Seattle supermarkets relative to King County supermarkets was for was for unprocessed or minimally processed foods, which experienced a nonsignificant decline of $0.02 per 100 kcal (SE = 0.04) from baseline to follow-up 1.

DD regression results for mean difference in price estimates over time between Seattle and King County supermarkets by nutrient density score, as defined by NRF_9.3_ quartiles, are shown in Table 3. Similar to the results of the analysis of effects across food groups and level of food processing, there was no significant minimum wage effect on the mean difference in price per 100 kcal in any NRF_9.3_ quartiles. The largest observed price change by NRF_9.3_ quartiles in Seattle supermarkets relative to King County supermarkets was for NRF_9.3_ quartile 4: “highly nutrient dense foods”, which experienced a nonsignificant decline of $0.04 per 100 kcal (SE = 0.07) from baseline to follow-up 3.

## 4. Discussion

This analysis expands on our prior work and contributes to the growing body of work evaluating the effect of minimum wage policies on food prices in four key ways. First, we evaluate changes in food prices per 100 kcal which tie changes in food prices more closely to how food purchasing decisions are likely to be made by price-sensitive individuals. Second, we evaluate an additional year of exposure to the Seattle Minimum Wage Ordinance as well as an additional increase in minimum wage. Third, this analysis presents the first evaluation of a $15 per hour minimum wage on local area supermarket food prices (Appendix Table A1). Fourth, we add an additional metric, the Nutrient Rich Food Index 9.3, as a way to further evaluate differential changes in food prices which are relevant to health. The results of this study lead us to conclude that local minimum wage increases did not result in changes in prices of food items sold at policy-affected supermarkets. There was also no evidence for differential effects of minimum wage increases on prices studied using multiple food quality metrics. These findings are important for two reasons. First low-wage earners and their families spend a larger share of their income on food [44]. Second, lower incomes have been linked to increased consumption of lower cost, energy dense foods of minimal nutritional value, and to poor health outcomes [24,36,45,46].

A net increase in wages could be used to purchase more nutritious food options, provided that the price of food stays constant. However, a recent, cross-sectional evaluation of the effect of the Seattle Minimum Wage Ordinance on low-wage jobs [47] found that the increase to $13/h reduced hours among low-wage jobs by 6–7% while hourly wages increase by 3%, leading to an overall reduction in income, on average. A follow-up, longitudinal analysis of low-wage workers [48] found that the most experienced half of workers (above median experience) saw earnings gains of approximately $19 per week while those with less experience (below median) saw little to no change, on average.

The present work adds to a limited but growing body of evidence evaluating the effect of minimum wage increases on grocery store prices in the United States [13,14,49,50,51]. The results of this evaluation and our prior studies [13,14] most closely align with the work of Ganapati and Weaver (2017) who found minimal evidence of pass-through effects on grocery store prices using national Nielsen retail price scanner data from 2005 to 2015 [49]. However, Leung (2018) also used national Nielsen retail price scanner data from 2006 to 2015 to evaluate the effect of federal, state, and local minimum wage increases and found that a 10% increase in minimum wage raised grocery store prices by 0.6–0.8%; however, there was a wide degree of effect heterogeneity by county-level and household-level socioeconomic status [50]. The estimated pass-through effect in poorer counties (defined by being below median Kaitz index) was greater than that of their rich counterparts and poorer households also reduced the intensity of their shopping following a minimum wage increase [50]. Another study by Renkin, et al (2017) found that a 20% increase in effective minimum wage led to a 0.4% increase in grocery store food prices in the first three months following the increase [51] in contrast to our results which did not observe any such immediate increase 1-month following Seattle’s minimum wage increase [13,14]. 

Further inference can be garnered from the evaluation of minimum wage increases and food prices in the fast-food and larger restaurant industries [10,11,52,53,54,55,56]. Seminal work by Katz and Kreuger (1992) and Card and Kreuger (1994) in examining federal minimum increases on fast-food restaurant food prices observed little effect that could be attributed to higher minimum wages [10,52]. MacDonald and Aaronson (2006) reported fast food price increases of 1.56% for a 10% increase in minimum wage, but also that item selection for price increases was related to other factors, such as a recent price reduction of the item [57]. Powers (2009) assessed the response of fast food prices to a 2004 state-level minimum wage increase in Illinois and found mixed evidence of an impact on fast food prices, however, the strongest price effects were observed in entrées [53]. Basker and Khan (2016) priced three menu items at New York City fast food restaurants throughout 1993–2014 to analyze price changes in response to five federal minimum wage increases that were implemented over the study period [54]. Their study found that a federal minimum wage increase of 33% (from $7.25/h to $10.10/h) would raise in fast food prices by 3%, an average increase of $0.10 per item [54]. However, the authors note that such an increase would likely be wholly offset by the corresponding increase in individual earnings among low-wage workers [54]. With respect to local minimum wage ordinances, Allegretto and Reich (2018) found that restaurant food prices in San Jose increased by 1.45% in response to a 25% increase in local minimum wage while Dube (2007) found a significant increase in prices of 6.2% at small and midsize fast food restaurants with table service in response to a 26% increase in minimum wage.

There are several reasons for the null findings we observed in our analyses. First, supermarkets may be using alternative channels to offset their increased costs, which are not consumer-facing. A report on the labor market effects of the Seattle minimum wage ordinance found that although minimum-wage workers experienced an increase in wages, a reduction in hours of approximately 35–50 min. per week per worker was also observed following the implementation of the policy [47]. Moreover, in a survey of Seattle employers, reducing hours or headcount was the second most common channel of adjustment across all for-profit industries following raising prices or adding fees [58]. Second, supermarkets may be raising the price of food items that were not included in the CPHN market basket, such as in-house prepared meals, salads, or bakery items [59]. Of the six supermarket chains surveyed, five had large prepared foods sections. Moreover, these items would require more labor by in-house employees. Third, supermarket prices may be more sensitive to national rather than local changes, particularly for large chain stores such as those included in this study. A market basket survey of Seattle supermarkets from 2002 to 2014 found that local-area food prices track closely with changes in the Consumer Price Index [38]. Another study found that within-chain price rigidity attenuated much of the observed effect of local minimum wage increases on retail prices [50]. Fourth, it is possible that employees at the sampled supermarket chains were earning at or above $15 per hour prior to the implementation of the ordinance and therefore the minimum wage increases implemented by the ordinance may not have been as binding for workers in this industry. Four out of the six supermarket chains were unionized [13,14] and larger firms, those with 500 or more employees globally, have been shown to be early adopters of mandated minimum wage increases [58]. However, we have previously shown [13,14], using administrative data, that twice as many grocery store jobs in Seattle and three times as many grocery store jobs (NAICS code 445110) in King County paid $11 an hour or less in the year prior to the passage of the Seattle minimum wage ordinance as compared with jobs in all other industries. Therefore, we believe it is likely that the minimum wage increases would have still lead to increased labor costs for these supermarket chains.

This study, however, was not without its limitations. First, this study did not collect data on food items prepared in-house, which requires more labor from supermarket employees. Second, prices reflect the lowest, non-discounted price and we are therefore unable to evaluate any sale or pricing strategies supermarkets may be employing to sway consumer choice. Third, while we attempted to evaluate food items by attributes most relevant to diet quality and, therefore, health, these results do not capture food purchasing habits of consumers nor do they speak to the diets or health of consumers. Fourth, Seattle’s unique economic conditions may limit the generalizability of our findings to other local minimum wage initiatives. Fifth, the timing of the implementation of the Seattle Minimum Wage Ordinance relative to the start of this funded evaluation did not allow for the collection of multiple pre-policy time points; therefore, we cannot determine with certainty that the food prices between Seattle and King County meet the parallel trends condition for difference-in-differences analysis. Future studies should consider evaluating the potential moderating role of region or other area-level socio-demographics on the effect of minimum wage on food prices. In addition, studies should consider using sales data to evaluate changes in consumer behavior as well as including prepared food items in their evaluation.

## 5. Conclusions

We find no evidence of changes in food prices overall or by food group, level of food processing, or nutrient density score, attributable to two years of exposure to the Seattle minimum wage ordinance and an increase in hourly wage to $13.50–$15.00 per hour. These null findings are encouraging as higher wages without a corresponding increase in the price of food may provide low-wage households with greater purchasing power for more healthy, nutritious foods. Given the growing trends of cities and states adopting higher minimum wages, other jurisdictions may find this market basket tool, as well as the categorization of food items, useful in evaluating the effect of wage increases on food prices in their area.

## Figures and Tables

**Table 1:** Overall and food group stratified linear difference-in-differences model results for the mean change in item-level price per 100 kcal across Seattle (‘intervention’) and King County (‘control’) stores and over time from March 2015 to May 2017, before and during implementation of Seattle’s minimum wage ordinance.

Mean Difference in Price Estimates Price per 100 kcal (in USD) (Robust Standard Errors)	Overall	Food Group						
		Cereals & Grains	Dairy	Fats & Oils	Fruits	Meats, Beans, Eggs, & Nuts	Sugar & Sweets	Vegetables
Seattle (relative to King County)	0.02	0.00	0.00	0.00	0.05	−0.01	0.03	0.05
	(0.082)	(0.020)	(0.039)	(0.018)	(0.139)	(0.065)	(0.140)	(0.174)
Follow-up 1 (relative to baseline)	0.00	0.00	−0.01	0.00	−0.04	−0.01	−0.08	0.07
	(0.017)	(0.009)	(0.006)	(0.003)	(0.057)	(0.006)	(0.075)	(0.043)
Follow-up 2 (relative to baseline)	−0.01	0.00	−0.01	0.00	0.01	−0.01	−0.10	0.02
	(0.014)	(0.013)	(0.010)	(0.005)	(0.062)	(0.016)	(0.086)	(0.026)
Follow-up 3 (relative to baseline)	0.03	−0.02 *	−0.03	0.00	0.05	0.00	−0.12	0.16 ***
	(0.019)	(0.011)	(0.016)	(0.005)	(0.069)	(0.023)	(0.087)	(0.034)
Seattle × Follow-up 1	−0.01	0.00	0.01	0.00	−0.03	0.00	−0.03	−0.03
	(0.026)	(0.011)	(0.012)	(0.004)	(0.070)	(0.008)	(0.132)	(0.063)
Seattle × Follow-up 2	0.00	0.00	0.00	0.00	−0.02	0.00	−0.03	0.00
	(0.019)	(0.019)	(0.014)	(0.007)	(0.082)	(0.022)	(0.147)	(0.036)
Seattle × Follow-up 3	0.00	−0.01	−0.01	0.01	−0.05	0.02	−0.02	−0.01
	(0.024)	(0.016)	(0.024)	(0.007)	(0.087)	(0.031)	(0.149)	(0.060)
Observations	4869	665	573	185	605	1424	286	1131
Number of stores	12	12	12	12	12	12	12	12
R^2^ within	0.0003	0.0031	0.0067	0.0139	0.0010	0.0006	0.0271	0.0018
R^2^ between	0.0025	0.2010	0.0000	0.0036	0.0069	0.0000	0.0000	0.0054
R^2^ overall	0.0003	0.0032	0.0055	0.0036	0.0012	0.0006	0.0253	0.0019

Notes: Baseline, March 2015 (1-month pre-policy enactment); follow-up 1, May 2015 (1-month post-policy enactment); follow-up 2, May 2016 (1-year post-policy enactment); follow-up 3 (2-years post-policy enactment). Robust standard errors are clustered by store. kcal = kilocalories. USD = United States dollars. *p* Values come from Wald tests. *** *p* < 0.001, * *p* < 0.05.

**Table 2:** Food processing category stratified linear difference-in-differences model results for the mean change in item-level price per 100 kcal across Seattle (‘intervention’) and King County (‘control’) stores and over time from March 2015 to May 2017, before and during implementation of Seattle’s minimum wage ordinance.

Mean Difference in Price Estimates Price per 100 kcal (in USD) (Robust Standard Errors)	Food Processing Category			
	Unprocessed or Minimally Processed Foods	Processed Culinary Ingredients	Processed Foods	Ultra-Processed Foods
Seattle (relative to King County)	0.03	−0.00	0.01	0.01
	(0.107)	(0.010)	(0.096)	(0.054)
Follow-up 1 (relative to baseline)	0.02	0.00	−0.03 *	−0.02
	(0.027)	(0.003)	(0.011)	(0.015)
Follow-up 2 (relative to baseline)	−0.01	0.01 ***	−0.01	−0.01
	(0.017)	(0.002)	(0.033)	(0.024)
Follow-up 3 (relative to baseline)	0.08 ***	0.00	−0.04	−0.04
	(0.022)	(0.002)	(0.038)	(0.024)
Seattle × Follow-up 1	−0.02	0.00	0.01	0.00
	(0.037)	(0.003)	(0.018)	(0.029)
Seattle × Follow-up 2	0.01	0.00	−0.01	−0.01
	(0.021)	(0.003)	(0.049)	(0.038)
Seattle × Follow-up 3	−0.01	0.00	0.01	0.00
	(0.034)	(0.003)	(0.045)	(0.039)
Observations	2,778	323	480	1,288
Number of stores	12	12	12	12
R^2^ within	0.0008	0.0107	0.0011	0.0049
R^2^ between	0.0052	0.0070	0.0006	0.0011
R^2^ overall	0.0009	0.0094	0.0010	0.0043

Notes: Baseline, March 2015 (1-month pre-policy enactment); follow-up 1, May 2015 (1-month post-policy enactment); follow-up 2, May 2016 (1-year post-policy enactment); follow-up 3 (2-years post-policy enactment). Robust standard errors are clustered by store. kcal = kilocalories. USD = United States dollars. *p* Values come from Wald tests. *** *p* < 0.001, * *p* < 0.05.

**Table 3:** Nutrient rich food index 9.3 (NRF 9.3) quartile stratified linear difference-in-differences model results for the mean change in item-level price per 100 kcal across Seattle (‘intervention’) and King County (‘control’) stores and time, from March 2015 to May 2017, following implementation of Seattle’s minimum wage ordinance.

Mean Difference in Price Estimates Price per 100 kcal (in USD) (Robust Standard Errors)	NRF 9.3 Quartile			
	Quartile 1: Least Nutrient Dense Foods	Quartile 2: Moderately Nutrient Dense Foods	Quartile 3: Nutrient Dense Foods	Quartile 4: Highly Nutrient Dense Foods
Seattle (relative to King County)	0.01	−0.00	0.01	0.06
	(0.051)	(0.033)	(0.099)	(0.169)
Follow-up 1 (relative to baseline)	−0.02	0.01	−0.02	0.05
	(0.016)	(0.006)	(0.029)	(0.039)
Follow-up 2 (relative to baseline)	−0.02	−0.01	0.00	0.01
	(0.021)	(0.009)	(0.025)	(0.030)
Follow-up 3 (relative to baseline)	−0.04	0.00	−0.01	0.18 ***
	(0.021)	(0.014)	(0.039)	(0.032)
Seattle × Follow-up 1	−0.00	−0.00	−0.01	−0.03
	(0.030)	(0.007)	(0.040)	(0.061)
Seattle × Follow-up 2	−0.01	0.01	0.00	0.00
	(0.035)	(0.017)	(0.034)	(0.037)
Seattle × Follow-up 3	0.00	0.00	0.01	−0.04
	(0.035)	(0.020)	(0.047)	(0.067)
Observations	1236	1194	1266	1173
Number of stores	12	12	12	12
R^2^ within	0.0048	0.0003	0.0001	0.0030
R^2^ between	0.0090	0.0002	0.0003	0.0071
R^2^ overall	0.0050	0.0002	0.0001	0.0032

Notes: Baseline, March 2015 (1-month pre-policy enactment); follow-up 1, May 2015 (1-month post-policy enactment); follow-up 2, May 2016 (1-year post-policy enactment); follow-up 3 (2-years post-policy enactment). Robust standard errors are clustered by store. kcal = kilocalories. USD = United States dollars. *p* Values come from Wald tests. *** *p* < 0.001.

## Appendix A

**Table A1:** Timeline of Seattle’s minimum wage increase during data collection.

Date of Data Collection	Minimum Wage Rate for Large Employers Not Paying Towards Employee Medical Benefits (in USD)	Minimum Wage Rate for Large Employers Paying Towards Employee Medical Benefits (in USD)	Time Point
March 2015	$9.47/h	$9.47/h	1-month pre-enactment
May 2015	$11.00/h	$11.00/h	1-month post-enactment
May 2016	$13.00/h	$12.50/h	1-year post-enactment
May 2017	$15.00/h	$13.50/h	2-year post-enactment

Notes: USD = United States dollars. Large employers are defined as 501 or more employees internationally. Two other phase-in schedules are possible for employers with 500 or fewer employees based on whether they contribute to employee benefits. For more information, please visit: https://www.seattle.gove/laborstandards/ordinances/minimum-wage.

**Table A2:** Food processing categorization based on the level of processing.

Food Processing Category	Defined as	Market Basket Examples
Unprocessed or minimally processed foods	Foods taken directly from nature; minimally processed to clean, pasteurize, freeze, or other processes that do not alter the composition	Rice, milk, apples, frozen turkey, broccoli (*n* = 59)
Processed culinary ingredients	Ingredients that can be added to unprocessed or minimally processed foods for flavor or seasoning used in the cooking process	Flour, butter, shortening, sugar (*n* = 12)
Processed foods	Unprocessed or minimally processed food that are processed or further processed, often with salt or oil, with the intent of extending shelf-life or altering palatability	Tortillas, tofu, canned salmon, canned corn (*n* = 10)
Ultra-processed foods	Foods that are highly processed with the intent of convenience and ready-to-eat/drink	Cookies, ice cream, salad dressing, sausages, cola, potato chips (*n* = 27)

Notes: NOVA food processing classification scheme taken from Martínez Steele, et al. (2016) [27]. Processed foods also include fermented alcoholic beverages; however, these were excluded from the present analysis.

**Table A3:** Nutrient density quartiles based on NRF_9.3_ score.

Nutrient Density Quartile	$\mathrm{Mean}\;{\mathrm{NRF}}_{9.3}\;\mathrm{Score}\;\pm \mathrm{SD}$	NRF_9.3_ Score Range	Example Foods
Quartile 1—Least nutrient dense foods	−13.6 ± 17.4	−51.6–8.8	Butter, cookies, bologna, potato chips, cola, cheese (*n* = 26)
Quartile 2—Moderately nutrient dense foods	20.2 ± 5.7	9.26–30.2	Potatoes, Turkey Eggs, steak, rice, bread (*n* = 25)
Quartile 3—Nutrient dense foods	56.8 ± 22.8	30.3–112.5	Peaches, chicken breast, beans, grapes, cereal, salmon, bananas (*n* = 27)
Quartile 4—Highly nutrient dense foods	232.3 ± 90.1	117.8–479.5	Green beans, spinach, grapefruit, sweet peppers, carrots, cantaloupe, asparagus, sweet potatoes (*n* = 25)

Notes: NRF = Nutrient Rich Foods index.

## References

[1] Autor D.H., Katz L.F., Kearney M.S. (2008). Trends in US Wage Inequality: Revising the Revisionists. Rev. Econ. Stat..

[2] Desilver D. (2017). 5 Facts about the Minimum Wage. http://www.pewresearch.org/fact-tank/2017/01/04/5-facts-about-the-minimum-wage/.

[3] UC Berkeley Labor Center Inventory of US City and County Minimum Wage Ordinances. http://laborcenter.berkeley.edu/minimum-wage-living-wage-resources/inventory-of-us-city-and-county-minimum-wage-ordinances/.

[4] Neumark D., Wascher W.L. (2010). The political economy of minimum wages. Minimum Wages.

[5] Office of the Mayor Edward B. Murray $15 Minimum Wage. http://murray.seattle.gov/minimumwage/.

[6] Aaronson D., French E. (2007). Product Market Evidence on the Employment Effects of the Minimum Wage. J. Labor Econ..

[7] Aaronson D. (2001). Price Pass-Through and the Minimum Wage. Rev. Econ. Stat..

[8] Aaronson D., French E., MacDonald J. (2008). The Minimum Wage, Restaurant Prices, and Labor Market Structure. J. Hum. Resour..

[9] Benner C., Jayaraman S. (2012). A Dime a Day: The Impact of the Miller/Harkin Minimum Wage Proposal on the Price of Food.

[10] Card D., Krueger A.B. (1994). Minimum wages and employment: A case study of the fast-food industry in New Jersey and Pennsylvania. Am. Econ. Rev..

[11] Ma J., Ghiselli R. (2016). The minimum wage, a competitive wage, and the price of a burger. Can competitive wages be offered in limited-service restaurants. J. Foodserv. Bus. Res..

[12] Lee C., Schluter G., O’Roark B., O’Roark B. (2000). Minimum wage and food prices: An analysis of price pass-through effects. Int. Food Agribus. Manag. Rev..

[13] Otten J.J., Buszkiewicz J., Tang W., Aggarwal A., Long M., Vigdor J., Drewnowski A. (2017). The Impact of a City-Level Minimum-Wage Policy on Supermarket Food Prices in Seattle-King County. Int. J. Environ. Res. Public Health.

[14] Spoden A.L., Buszkiewicz J.H., Drewnowski A., Long M.C., Otten J.J. (2018). Seattle’s minimum wage ordinance did not affect supermarket food prices by food processing category. Public Health Nutr..

[15] Rehm C.D., Monsivais P., Drewnowski A. (2015). Relation between diet cost and Healthy Eating Index 2010 scores among adults in the United States 2007–2010. Prev. Med..

[16] Carlson A., Frazão E. (2014). Food costs, diet quality and energy balance in the United States. Physiol. Behav..

[17] Drewnowski A., Darmon N. (2005). The economics of obesity: Dietary energy density and energy cost. Am. J. Clin. Nutr..

[18] Williams P.L., Johnson C.P., Kratzmann M.L.V., Johnson C.S.J., Anderson B.J., Chenhall C. (2006). Can households earning minimum wage in Nova Scotia afford a nutritious diet?. Can. J. Public Health.

[19] Monsivais P., Drewnowski A. (2009). Lower-energy-density diets are associated with higher monetary costs per kilocalorie and are consumed by women of higher socioeconomic status. J. Am. Diet. Assoc..

[20] Wright A., Smith K.E., Hellowell M. (2017). Policy lessons from health taxes: A systematic review of empirical studies. BMC Public Health..

[21] Powell L.M., Chriqui J.F., Khan T., Wada R., Chaloupka F.J. (2013). Assessing the potential effectiveness of food and beverage taxes and subsidies for improving public health: A systematic review of prices, demand and body weight outcomes. Obes. Rev..

[22] Olsho L.E., Klerman J.A., Wilde P.E., Bartlett S. (2016). Financial incentives increase fruit and vegetable intake among Supplemental Nutrition Assistance Program participants: A randomized controlled trial of the USDA Healthy Incentives Pilot. Am. J. Clin. Nutr..

[23] Drewnowski A., Kawachi I. (2015). Diets and Health: How Food Decisions Are Shaped by Biology, Economics, Geography, and Social Interactions. Big Data.

[24] Darmon N., Drewnowski A. (2008). Does social class predict diet quality?. Am. J. Clin..

[25] Centers for Disease Control and Prevention (2018). State Indicator Report on Fruits and Vegetables.

[26] French S.A., Rydell S.A., Mitchell N.R., Michael Oakes J., Elbel B., Harnack L. (2017). Financial incentives and purchase restrictions in a food benefit program affect the types of foods and beverages purchased: Results from a randomized trial. Int. J. Behav. Nutr. Phys. Act..

[27] Martínez Steele E., Baraldi L.G., da Costa Louzada M.L., Moubarac J.-C., Mozaffarian D., Monteiro C.A. (2016). Ultra-processed foods and added sugars in the US diet: Evidence from a nationally representative cross-sectional study. BMJ Open.

[28] Rauber F., da Costa Louzada M.L., Steele E.M., Millett C., Monteiro C.A., Levy R.B. (2018). Ultra-Processed Food Consumption and Chronic Non-Communicable Diseases-Related Dietary Nutrient Profile in the UK (2008–2014). Nutrients.

[29] Nardocci M., Leclerc B.-S., Louzada M.-L., Monteiro C.A., Batal M., Moubarac J.-C. (2018). Consumption of ultra-processed foods and obesity in Canada. Can. J. Public Health.

[30] Monteiro C.A., Cannon G., Moubarac J.-C., Levy R.B., Louzada M.L.C., Jaime P.C. (2018). The UN Decade of Nutrition, the NOVA food classification and the trouble with ultra-processing. Public Health Nutr..

[31] Machado P.P., Claro R.M., Canella D.S., Sarti F.M., Levy R.B. (2017). Price and convenience: The influence of supermarkets on consumption of ultra-processed foods and beverages in Brazil. Appetite.

[32] Drewnowski A., Fulgoni V. (2008). Nutrient profiling of foods: Creating a nutrient-rich food index. Nutr. Rev..

[33] The Seattle Minimum Wage Study Team (2016). Early Evidence on the Impact of Seattle’s Minimum Wage Ordinance.

[34] City of Seattle Office of Labor Standards (2016). Minimum Wage Ordinance. http://www.seattle.gov/Documents/Departments/LaborStandards/OLS-FactSheets-MWO.pdf.

[35] The Seattle Minimum Wage Study Team (2017). The Seattle Minimum Wage Ordinance October 2017 Update: Report on Employer Adjustments, Worker Experiences, and Price Changes.

[36] Drewnowski A., Aggarwal A., Hurvitz P.M., Monsivais P., Moudon A.V. (2012). Obesity and supermarket access: Proximity or price?. Am. J. Public Health.

[37] United States Department of Agriculture Agricultural Research Service (2018). Food Composition Databases. https://ndb.nal.usda.gov/ndb/.

[38] Freeman K.O. (2014). The Cost of Healthy Foods in Seattle, WA: Price Trends from 2004–2014. Ph.D. Thesis.

[39] United States Department of Agriculture (2012). Quarterly Food-at-Home Price Database. http://www.ers.usda.gov/data-products/quarterly-food-at-home-price-database/documentation.aspx.

[40] Fulgoni V.L., Keast D.R., Drewnowski A. (2009). Development and Validation of the Nutrient-Rich Foods Index: A Tool to Measure Nutritional Quality of Foods. J. Nutr..

[41] Drewnowski A. (2009). Defining Nutrient Density: Development and Validation of the Nutrient Rich Foods Index. J. Am. Coll. Nutr..

[42] United States Department of Agriculture Agricultural Research Service (2018). USDA National Nutrient Database for Standard Reference, Legacy. https://ndb.nal.usda.gov/ndb/.

[43] StataCorp (2015). Stata Statistical Software.

[44] Holcomb R.B., Park J.L., Capps O. (1995). Revisiting Engel’s Law: Examining Expenditure Patterns for Food at Home and Away from Home. J. Food Distrib. Res..

[45] Monsivais P., Drewnowski A. (2007). The rising cost of low-energy-density foods. J. Am. Diet. Assoc..

[46] Drewnowski A., Specter S. (2004). Poverty and obesity: The role of energy density and energy costs. Am. J. Clin..

[47] Jardim E., Long M., Plotnick R., van Inwegen E., Vigdor J., Wething H. (2018). Minimum Wage Increases, Wages, and Low-Wage Employment: Evidence from Seattle.

[48] Jardim E., Long M., Plotnick R., van Inwegen E., Vigdor J., Wething H. (2018). Minimum Wage Increases and Individual Employment Trajectories.

[49] Ganapati S., Weaver J. (2017). Minimum Wage and Retail Price Pass-Through: Evidence and Estimates from Consumption Data. SSRN.

[50] Leung J. (2018). Minimum Wage and Real Wage Inequality: Evidence from Pass-Through to Retail Prices. SSRN.

[51] Renkin T., Montialoux C., Siegenthaler M. (2017). The Pass-through of Minimum Wages into US Retail Prices: Evidence from Supermarket Scanner Data. https://economics.ceu.edu/sites/economics.ceu.edu/files/attachment/event/1118/tobiasrenkinjmp.pdf.

[52] Katz L.F., Krueger A. (1992). The Effect of the Minimum Wage on the Fast-Food Industry. Ind. Labor Relat. Rev..

[53] Powers E.T. (2009). The Impact of Minimum-Wage Increases: Evidence from Fast-food Establishments in Illinois and Indiana. J. Labor Res..

[54] Basker E., Khan M.T. (2016). Does the Minimum Wage Bite into Fast-Food Prices?. J. Labor Res..

[55] Dube A., Naidu S., Reich M. (2007). The Economic Effects of a Citywide Minimum Wage. ILR Rev..

[56] Allegretto S., Reich M. (2018). Are Local Minimum Wages Absorbed by Price Increases? Estimates from Internet-Based Restaurant Menus. ILR Rev..

[57] MacDonald J.M., Aaronson D. (2006). How Firms Construct Price Changes: Evidence from Restaurant Responses to Increased Minimum Wages. Am. J. Agric. Econ..

[58] Romich J.L., Allard S.W., Obara E.E., Althauser A.K., Buszkiewicz J.H. (2018). Employer Responses to a City-Level Minimum Wage Mandate: Early Evidence from Seattle. Urban Aff. Rev..

[59] Wilde P. (2018). Food Policy in the United States: An Introduction.

